# Accessing Meals on Wheels: A qualitative study exploring the experiences of service users and people who refer them to the service

**DOI:** 10.1111/hex.13943

**Published:** 2023-12-19

**Authors:** Angeliki Papadaki, Mary Wakeham, Becky Ali, Miranda Elaine Glynis Armstrong, Paul Willis, Ailsa Cameron

**Affiliations:** ^1^ Centre for Exercise, Nutrition and Health Sciences, School for Policy Studies University of Bristol Bristol UK; ^2^ Centre for Research in Health and Social Care University of Bristol Bristol UK; ^3^ Population Health Sciences, Bristol Medical School University of Bristol Bristol UK

**Keywords:** carers, community meals, home‐delivered meals, Meals on Wheels, qualitative research, referral

## Abstract

**Aims:**

This study aimed to explore the perceptions of Meals on Wheels (MoWs) service users (SUs), and people who refer them to MoWs (‘referrers’), with accessing and commencing the service in England, the barriers that might hinder service uptake, and what information would be valued when considering accessing the service.

**Methods:**

Semistructured interviews were conducted in May–July 2022 with seven SUs and 21 referrers, recruited from four MoWs providers across England. Data were analysed using inductive thematic analysis.

**Results:**

Participants indicated various pathways into the service, but referrers (family members) were more likely to be the ones enquiring about, and commencing, MoWs for SUs. Once an enquiry about MoWs had been made, the service was perceived as straightforward to set up. However, existing preconceptions and stereotypes were perceived to act as barriers to accessing MoWs. Information that participants deemed important to have available when deciding on whether to access MoWs related to the meals, the specific services provided, the reliability and flexibility of delivery and the cost of services.

**Conclusion:**

These findings could inform MoWs service providers' public awareness strategies about MoWs, to facilitate referrals to the service for adults with care and support needs.

**Patient or Public Contribution:**

An advisory group of people with lived experience of MoWs (users of the service and their family referrers) extensively discussed the findings of the research and advised on the implications and future dissemination steps.

## 1 INTRODUCTION

Meals on Wheels (MoWs) is a crucial service delivering food to older adults, and adults with care and support needs, who are unable to leave their home and/or might not be able to acquire and prepare their own meals. Consistent evidence suggests that MoWs improve nutrient status in older adults,^1^, ^2^ and offer benefits that extend beyond nutrition, including the provision of wellbeing checks, social interaction, decreased rates of isolation and improved quality of life.^3^, ^4^ The use of MoWs has also been linked to a decreased need for residential care, by helping service users (SUs) continue living in their homes and communities for longer.^5^, ^6^ These beneficial outcomes achieved by MoWs present significant preventative measures that contribute to the wellbeing of adults with care and support needs. MoWs services will undoubtedly become essential with increasing rates of an ageing population,^3^ and adults living with multiple morbidities and complex needs.^7^, ^8^

In England, MoWs have been traditionally provided by local councils with responsibilities for adult social care, which deliver a daily hot meal, but also chilled and/or frozen meals, either in addition to the hot meal provision, or as a substitute.^9^, ^10^ However, reductions in central government grants to councils in 2009/2010 meant that 54,795 fewer individuals accessed MoWs in the following 3 years,^11^ while only 42% of Councils provided MoWs in 2018, with 24% terminating services since 2014.^9^ Although providers from the private or nonprofit sector and social enterprises have filled gaps in MoWs provision when councils have withdrawn their MoWs services,^9^ many individuals are purportedly not aware that the service exists.^12^ The lack of awareness that MoWs exist has also been documented by studies in Ireland^13^ and New Zealand,^14^ potentially contributing to poor referral systems and reduced access to MoWs by adults with care and support needs who could benefit from the service. Collectively, these findings make it essential to explore the experiences of users of MoWs with accessing the service.

Several pathways into accessing or finding out about MoWs services have been reported in the international literature, including at the point of hospital discharge in Ireland,^15^ via an assessment team, general practitioners and self‐/family referrals in New Zealand,^14^ and via carers (e.g., friends/family) and self‐referrals, in addition to referrals from hospitals, general practitioners, aged care assessment teams, home and community care services and community‐based disability services in Australia.^16^ Nevertheless, several barriers to accessing MoWs have been documented. For example, a recent qualitative study amongst MoWs SUs in Ireland found a lack of information on what MoWs services entail, lack of clarity about eligibility criteria and referral pathways and lack of knowledge about commencing the service.^13^ Confusion about eligibility criteria was also reported to be a barrier to using the service amongst older adults in New Zealand, while lack of knowledge about the type of support offered (i.e., long‐term or temporary) was considered a barrier to referring patients to MoWs amongst health professionals.^14^

To our knowledge, there is no research exploring the perceptions of MoWs SUs, and people who support MoWs recipients and/or refer them to MoWs (‘referrers’), with accessing and commencing the service in England. In addition, no research has explored what information MoWs SUs and their referrers would value when considering accessing MoWs services. The aim of this small‐scale study was therefore to explore these dimensions of MoWs services, in light of the experience of MoWs SUs and referrers, recruited from different areas in England.

## 2 METHODS

This study uses the methods detailed in a recent publication.^17^ Study reporting followed the Consolidated Criteria for Reporting Qualitative Research guidelines^18^ (Table S1). The study was approved by institutional/local review board. Participants were provided with detailed verbal and written information about the study, gave informed consent before data collection and received a £20 gift voucher as a token of appreciation.

### 2.1 Participants

Participants were current MoWs SUs, and referrers of current MoWs SUs, recruited from four service providers in England (one local authority, one social enterprise, a private sector provider and a family business). These MoWs service providers were based in the North West, South West, South East and East Midlands; these regions of England were purposively selected according to geographic location (urban and semiurban). The managers of MoWs services acted as gatekeepers for participant recruitment. This involved delivering a study invitation and participant information sheet to SUs via MoWs delivery drivers, during normal meal delivery times, and e‐mailing the study invitation and participant information sheet to people who had referred a current MoWs SU to the service.^17^

### 2.2 Data collection

Semistructured interviews, lasting 18–49 min, were conducted via telephone in May–July 2022 and audio recorded. Two interview guides were developed to explore the experiences of MoWs SUs, and referrers (Tables S2 and S3). The guides were not piloted, but were adapted from those used in recent research amongst MoWs service providers in England.^12^ In short, the interviews explored participants' experiences with accessing and commencing MoWs, and the information about MoWs they sought when initially enquiring about the service (the focus of the current paper). Additional topics of discussion (explored in a separate publication), included questions about the meals received, the perceived benefits of using MoWs, whether participants' experiences with the service had changed during the COVID‐19 pandemic, and perceptions around the need to improve MoWs services.^17^

All interviews were conducted by the second author, an experienced qualitative researcher. No relationship had been established between the interviewer and participants before study commencement. Field notes were kept to verify responses at transcription, and a summary of main points discussed was provided to participants at the end of the interviews to confirm accuracy of responses.^19^ Interviews were transcribed verbatim and anonymised; transcripts were compared with recordings and field notes to verify credibility. Data collection and analysis proceeded in parallel.

### 2.3 Data analysis

Data were analysed using thematic analysis,^20^ informed by a phenomenological approach.^21^ The second author read through all transcripts and coded them inductively, which involved initial coding of the data into broad codes. Data were then analysed line‐by‐line to create specific codes. The third author independently coded four transcripts to ensure rigour of the process.^19^ Discrepancies were discussed and the coding process was further refined; the second author then used this codebook to code all transcripts, noting any new codes. Using NVivo (version 12.0, QSR), the codes were organised into themes and subthemes, and further reviewed by the team to ensure coherence within and across themes.^22^ Findings from the two qualitative data sources (SUs and referrers) were combined to demonstrate the emergent themes and subthemes,^17^ which are illustrated with representative quotations from participants (indicated as SU 1–7/referrer [R] 1–21). Additional quotations are provided in Table S4.

## 3 RESULTS

Seven SUs (mean age = 87 years; *n* = 5 females) and 21 referrers (*n* = 18 females) took part in interviews (Table 1). The majority of referrers (*n* = 15) had referred their parent(s) to the service, followed by a sibling (*n* = 2). Referrers who were family members were the most reported people to have commenced MoWs for a SU, followed by social care/support workers employed via social care agencies. For SUs, the most frequently reported reasons for accessing MoWs were mobility challenges and inability to perform everyday activities (*n* = 4). The most common reasons for commencing MoWs for a SU, as reported by referrers, were cognitive decline (*n* = 9) and mobility challenges (*n* = 3). Findings are presented under two main themes (Table 2).

**Table 1:** Participant characteristics (*n*, %).

	Meals on Wheels service users (*n* = 7)	Meals on Wheels referrers (*n* = 21)
Area of England		
South West	4 (57.1)	7 (33.3)
North West	3 (42.9)	3 (14.3)
South East	0 (0.0)	4 (19.0)
East Midlands	0 (0.0)	7 (33.3)
Sex of participant		
Male	2 (28.6)	3 (14.3)
Female	5 (71.4)	18 (85.7)
Sex of service user referred		
Male	–	11 (52.4)
Female	–	9 (42.9)
Male and female (two service users)	–	1 (4.8)
Relationship of referrer to service user	–	
Niece/nephew		1 (4.8)
Child/step child		15 (71.4)
Grandchild		1 (4.8)
Sibling		2 (9.5)
Carer		1 (4.8)
Power of attorney		1 (4.8)
Age of service user (years)^a^	86.8 (8.8, 76–94)	83.9 (10.3, 57–94)
Who commenced the service		
Referrer	3 (42.9)	20 (95.2)
Social care/support worker	4 (57.1)	1 (4.8)
Reason for commencing the service		
Blindness	1 (14.3)	2 (9.6)
Dementia	0 (0.0)	9 (42.9)
Hip fracture/knee replacement	1 (14.3)	1 (4.8)
Following hospital discharge	0 (0.0)	2 (9.6)
Inability to perform everyday activities due to ageing	2 (28.6)	0 (0.0)
Mobility challenges	2 (28.6)	3 (14.3)
Learning disability	0 (0.0)	1 (4.8)
Stroke	1 (14.3)	1 (4.8)
Self‐neglect	0 (0.0)	1 (4.8)
Mental health condition	0 (0.0)	1 (4.8)
Who pays for Meals on Wheels		
Service user	7 (100.0)	21 (100.0)

^a^ Numbers represent mean (standard deviation, range).

**Table 2:** Themes and subthemes resulting from the thematic analysis.

Theme	Subtheme
Accessing and commencing the service	− Referrals to Meals on Wheels − Knowledge of the MoWs concept and commencing the service − Barriers to accessing Meals on Wheels
Information valued when enquiring about Meals on Wheels for the first time	− Information relating to the meals − Information relating to the specific services provided

## 4 ACCESSING AND COMMENCING THE SERVICE

### 4.1 Referrals to MoWs

Various routes of professional referral to the MoWs service were reported by participants, including via adult social care needs assessments, support/social workers and domiciliary care workers. Often, it was these individuals who commenced MoWs for SUs.

I had a care needs assessment, carried out by a social worker from the Council. She came and did a thorough investigation into what my care needs were… She said she would do it for me, and she did. (SU3)

Sometimes, however, support workers signposted family members to MoWs, and they made the actual referral.

And I think their social workers were involved, so I think they suggested it as well and then I actually followed it up. (R17)

Many referrers highlighted that they were the ones who enquired about, and commenced, MoWs for SUs, by conducting internet searches or phoning the local Council. Even though self‐referrals were possible, one referrer highlighted that the family member they referred would not have accessed MoWs on their own.

He wouldn't have gone off his own backside to do it. It was only that I thought that perhaps it's a good—‘Well let's give it a go dad’ and he was like, ‘Oh yeah, let's give that a go’. But yeah, I'm not sure that he would have thought about that himself. (R15)

### 4.2 Knowledge of the MoWs concept and commencing the service

Some participants perceived that although ‘I didn't know what format (the service) was in nowadays’ (R10), and ‘even if it's not called Meals on Wheels anymore’ (R3), MoWs was a well‐known concept to them; some were aware of the service via word of mouth and the media, while others through previous involvement of family members who had been working for the service, or had been recipients of MoWs in the past. Despite historical knowledge of the service; however, one participant highlighted that any knowledge about MoWs does not ‘register’ until one is actually in need of the service.

I mean, I'm sure I've heard the name ‘Meals on Wheels’ before, but until you really experience it, you don't really know what it's about. (R12)

Once they had enquired about MoWs, participants reported that the process of commencing the service was ‘really straightforward actually’ (SU1). In addition to the information that had already been provided to them verbally or via the internet, commencing MoWs included receiving written materials and packs with information containing contact details and explanations of the process of ordering meals.

… It was really clear cut, as to the rules that they work by. (SU2)

### 4.3 Barriers to accessing MoWs

Several existing preconceptions were perceived to act as a barrier, or to result in initial resistance to accessing MoWs services. For example, despite participants highlighting the quality and tastiness of the meals, they perceived a stereotype still exists about the food quality and that MoWs might remind people of ‘school meals’.

My neighbour, I introduced her to it… I said, ‘Well, why don't you get yourself… meals on wheels?’ and she said that it reminded her of school meals… I know a lot of people that are a bit snobby about this, but I have really good meals, tasty meals… (SU3)

In addition, referrers highlighted that MoWs are marketed for, or are thought to be *‘*for old people’ (R4). This might prevent *‘*anybody who has a need for a hot meal who physically can't do it themselves’ (R6) to access the service, but also might incline older adults who might be in need of the service to be resistant to using MoWs.

She didn't consider herself old enough to be receiving it even though she's 79 at the moment, nearly 80. (R16)

This might relate to feelings of pride or wanting to remain as independent as possible, with one referrer highlighting:

It's quite difficult to get the elderly to do this because, ‘They don't need the help apparently’, as I'm constantly being told. (R21)

Interestingly, one participant reported how they addressed this initial resistance by ‘I ordered at first for two or three weeks, meals for myself, Dad and my sister. So, all three of us sat down and had the meals, and it was a way of introducing Dad’ (R9).

## 5 INFORMATION VALUED WHEN ENQUIRING ABOUT MOWS FOR THE FIRST TIME

### 5.1 Information relating to the meals

When asked what information they sought the first time they considered accessing the service, or the first time they enquired about the service, participants reported several dimensions related to the meals. These included the quality, taste and nutritional value of the meals. For example, it was deemed important to have information on ‘what type of meals are provided’ (SU1) and whether users of the service would be receiving a ‘varied and balanced menu’ (R10). In addition, participants wanted to know whether the meal was hot, and/or if any preparation would be required before the meal was consumed.

What I wanted to ensure was that he got a cooked meal that he just had to eat. Because at that time, I suspected he couldn't be bothered preparing it. (R1)

Information on the types of meals provided, the meal options available (e.g., ‘I think the most important thing for me would be in terms of the choices’, R12), and whether the service caters for personal preferences or individual needs (e.g., ‘because my dad is a very fussy eater’, R17), was also considered important to be provided. For instance, it was important for participants to know whether the meals were suitable for people with mobility challenges.

He simply cannot use the oven. I had to make sure that their meals were microwaveable, let's put it that way. (R7)

### 5.2 Information relating to the specific services provided

For referrers, finding out whether wellbeing checks were provided, and that they would be contacted if a safety concern was identified, or ‘if they felt there was something different that they weren't happy with’ (R5), was deemed essential.

Touch wood, if anything was wrong, that they would also obviously contact us, where other services for home meal delivery, they all give you a week's worth of food in 1 day. You haven't got someone daily coming in. (R8)

Establishing that MoWs provided daily social contact was indeed also considered important.

(I wanted to know) it wasn't a case of just turning up, dumping it on the doorstep and going away, that would've been completely inappropriate. (R19)

Another dimension of MoWs that participants would value information on was the reliability and continuity of the service (e.g., ‘I just wanted to know… whether they provided it regularly without interruption and they have done that’, SU1), including the time of meal delivery, and also who delivers the meals. For example, ensuring regularity was deemed particularly important for SUs with cognitive decline.

So one of the things that was quite important is the time they turned up was a regular time. It also was very important they'd have, if not the same person every day, at least regulars if you see what I mean? (R21)

In addition, some referrers highlighted the value of knowing whether the service could be tailored to SUs' needs.

So I needed to know that someone would go into the house, put (the meal) out for her and make sure she was happy and make sure she had a drink. These are the areas that cause the biggest problems. (R13)

Further, the cost of MoWs and the available payment options (e.g. whether payment could be arranged on a pay‐as‐you‐go basis or via direct debit) was important for participants to know before deciding whether to commence MoWs. One user of the service, in particular, suggested that the cost of MoWs would be important to consider ‘because of the cost of living nowadays’ (SU6). Finally, some participants wanted to know how flexible the service would be, for example with cancellations, the number of days per week the meals would be delivered, or the duration a SU would need the service.

Because if it was going to be temporary, I wanted something easy to set up so that we could cancel it if we wanted to. I wanted to check about‐ do you need to have every day, could you take some days off, so that was important to us. Yeah, I think probably the flexibility of it really was probably important to start off with. (R15)

## 6 DISCUSSION

This study aimed to explore the experiences of SUs, and people who refer them to the service, with accessing and commencing MoWs in England, as well as what information would be valued when considering accessing, and enquiring about, MoWs. Our findings highlight that despite the variety of MoWs referral routes identified, referrers who are family members are perceived as most likely to enquire about, and commence MoWs, for adults with care and support needs. The service was perceived as easy to commence, but several preconceptions were identified that could hinder MoWs uptake. Several types of information were also identified, which SUs and referrers deemed as important to have available before making decisions about commencing MoWs for them and/or their families, respectively. These findings present a crucial formative evidence base, which could inform providers' public awareness strategies about MoWs services.

Participants highlighted several referral pathways, or ways of being signposted to MoWs services. Professional referrals included those made by social workers through social care needs assessments (sometimes initiated because a SU had been hospitalised), as well as by other support workers and carers employed by social care agencies. These routes into MoWs services are also reported in earlier international studies.^14^, ^15^, ^16^ Participating referrers, however, reported that they were the ones most likely to refer and commence MoWs for a family member. In addition, none of the users of the service who participated in the study reported that they had self‐referred. Although self‐referrals were possible, some referrers perceived that their family members would not have accessed MoWs if they had not commenced the service for them, potentially due to the barriers to accessing MoWs highlighted in the current study. Self‐referrals to MoWs, and/or referrals to MoWs from family and friends, have been documented as a highly prevalent pathway into the service in studies in New Zealand^14^ and Australia.^16^ In fact, a case study audit of a MoWs provider in Australia found a 17% increase in these referral routes in a period of 4 years (2008–2012), with a parallel decrease in MoWs referrals from hospitals and care community services.^16^ Neither the findings of this earlier study, nor those from the current study, portray a representative picture of referral patterns. Nevertheless, they collectively indicate that advocacy activities for MoWs services should potentially target general practitioners, hospital discharge teams and social and community carers/workers. As social workers and healthcare professionals often advise on whether an adult with care and support needs should access care and support services,^23^ highlighting the availability of MoWs services and raising awareness of the benefits of MoWs amongst care practitioners could increase professional referrals. This could support more adults with care needs to continue living in their homes and communities for longer, potentially saving social care services and the National Health Service millions of pounds, similar to projections in the United States.^6^ Councils with adult social care responsibilities could also ensure that assessments of food needs, an important aspect of the Adult Social Care Outcomes Framework in the United Kingdom,^24^ are included in their social care assessments, so that every adult with care and support needs who could benefit from the service is referred to it.

Several preconceptions and stereotypes surrounding MoWs were reported by participants, which could present barriers to accessing the service or result in initial resistance to access services. Referrers, in particular, highlighted the ‘stigma’ of MoWs use being linked to older age. This could prevent younger adults, who could benefit from the service because they cannot prepare their own meals due to disabilities, to enquire about it, as also suggested by a recent study in Ireland.^13^ As reported by some referrers in the current study, older adults could feel reluctant to access MoWs because they might find it hard to come to terms with their loss of independence, and associated questions about their identity and self‐worth, or the fact that they might require help. These perceptions have been corroborated by earlier research.^15^, ^25^ Interestingly, another source of stigma reported in the literature relates to perceptions that using MoWs is similar to accepting charity, and is linked to feelings of loss of pride^13^, ^14^, ^15^; we did not find this in our study. Overall, MoWs providers could address preconceptions and stereotypes surrounding age‐related eligibility criteria by highlighting to potential SUs, and their referrers, that MoWs is for any individual who cannot prepare their own meals, irrespective of age. In addition, it has been suggested that older adults might be reluctant to access services if they consider their quality to be low.^25^ As a participating user of the service in the current study reported that adults with care and support needs might not be commencing MoWs because the meals delivered remind people of ‘school meals’, and some referrers suggested stigmatism associated with quality of the food, MoWs service providers should consider highlighting the tastiness, and quality, of the meals on offer, as reported in earlier research.^17^ This could be achieved, for example, by including SU testimonials about the quality of the meals on their websites and publicity materials.

A recent study amongst MoWs providers in South West England suggested that many people who could benefit from the service do not know that MoWs exist.^12^ Participants in the current study had generally heard of the MoWs service before they accessed it. Nevertheless, referrers highlighted that people do not register such information until they actually need the service for themselves or a family member. Indeed, lack of knowledge about the service has been suggested to be the main barrier to accepting MoWs amongst health professionals and older adults in New Zealand.^14^ These findings suggest that MoWs providers should consider enhancing their publicity activities to ensure wide awareness of the service's existence, in addition to ensuring appropriate information is easily available, and accessible, to adults with care and support needs, and anyone who might refer an adult with care and support needs to the service, early on in the process. The latter is legislated by the Care Act 2014, which specifies that information on social care services that prevent, or delay the need for residential care, should be accessible by those who might be in need of such services.^26^

Earlier research in New Zealand has suggested that health professionals are not aware of specific aspects of MoWs, such as the cost of services, nutritional value of the meals, and the number of meals that users of the service could receive, potentially hindering referrals to the service.^14^ To our knowledge, no study has examined awareness of MoWs, and specific aspects of the service, amongst health and social care professionals in England, and therefore these findings should be corroborated by future research in this country. However, the current study highlighted that MoWs SUs and referrers would value knowing this information early on in the process of accessing the service. In addition, participants in the current study perceived that having information about additional services provided by MoWs, such as wellbeing checks and help with plating up the meal, as well as information on eligibility criteria, is important to aid decisions around accessing and commencing MoWs services for themselves or their family members. Therefore, MoWs providers should seek to enhance their online information provision to facilitate informed decision‐making by anyone wishing to enquire about the service. MoWs providers could also be encouraged to provide the calorie information of the meals on their websites, similar to recent UK legislation that requires restaurants to display this information on their menus.^27^

## 7 STRENGTHS AND LIMITATIONS

To our knowledge, this is the first study to explore the perceptions and experiences of MoWs SUs and referrers, with how they access and commence the service in England, and the information they would value when enquiring about accessing the service. As such, our findings add to the international evidence base on MoWs referral pathways, perceived barriers to MoWs uptake, and service awareness, but also provide novel insights on the information required to aid decision making when considering accessing MoWs, which could be of value to community nutrition and social care practitioners, as well as MoWs service managers, worldwide. Nevertheless, this was a small‐scale study, which limits the transferability of the findings. For example, we did not collect data on ethnicity, and therefore the findings might not represent the experiences of the diverse population of England who might be using the service. Nevertheless, a variety of reasons for accessing MoWs was reported by participants, and we recruited through four different types of MoWs providers from diverse areas in England, which contributed to the reporting of diverse experiences. In addition, the number of SUs recruited into the study was small, and recruited from only two of the four participating MoWs providers. Despite not aiming to conduct a formal comparison of views between SUs and referrers, interrogation of the data did not reveal any major differences in experiences with accessing the service between the two groups. Nevertheless, future studies should identify feasible and acceptable recruitment methods to encourage users of MoWs services to participate in similar research.

## 8 CONCLUSIONS AND IMPLICATIONS

This study identified several MoWs referral pathways in England, with family members perceived as the individuals to most likely commence MoWs for users of the service. Once they had enquired about the service, commencing MoWs was perceived as straightforward, but several preconceptions were identified that were thought might hinder MoWs uptake. Furthermore, information about several aspects of the meals and the service were perceived to be important to have available, before people who are interested in accessing the service decide whether to commence MoWs. Collectively, our findings indicate that MoWs service providers should seek to enhance awareness of the service and its wider preventative value. This could be done via appropriate framing of publicity messages and awareness‐raising activities, which will address identified stereotypes surrounding MoWs use, and provide the information valued to facilitate referrals to the service. This could contribute to MoWs being accessed by all individuals who live in the community who could benefit from the service.

## AUTHOR CONTRIBUTIONS

Angeliki Papadaki conceived the study, with input from Paul Willis, Ailsa Cameron and Miranda Elaine Glynis Armstrong. Mary Wakeham collected the data. Mary Wakeham, Becky Ali and Angeliki Papadaki analysed the data, with input from all co‐authors. Angeliki Papadaki led the drafting of the manuscript. All authors provided critical input, reviewed the manuscript for important content, take responsibility for the contents of this article and approved the final version submitted for publication. The manuscript has been made available on a pre‐print server and can be found here: https://www.preprints.org/manuscript/202309.0310/v1, but has not been published elsewhere.

## CONFLICT OF INTEREST STATEMENT

The authors declare no conflict of interest.

## ETHICS STATEMENT

The study was approved by the University of Bristol, Faculty of Social Sciences and Law Research Ethics Committee (reference 0170). Participants were provided with detailed verbal and written information about the study, and gave informed consent before data collection.

## Supporting information

Supporting information.

## Data Availability

The interview guides used to collect data, as well as processed data, are available in the supplementary material of this article. Other data that support the findings of this study are available from the corresponding author upon reasonable request.
