# Supplementary material for: Accessing Meals on Wheels: A qualitative study exploring the experiences of service users and people who refer them to the service
Source: Health Expect. 2023 Dec 19;27(1):e13943. doi: 10.1111/hex.13943 (PMC10729527; doi:10.1111/hex.13943)
Supplement: Supplementary file 1 — Supporting information. [file HEX-27-e13943-s001.docx]

**Table S1:** COREQ (Consolidated criteria for reporting qualitative studies) checklist

| **No** | **Item** | **Guide questions/description** | **Page** |
| --- | --- | --- | --- |
| **Domain 1: Research team and reflexivity** | | | |
| **Personal Characteristics** | | | |
| 1. | Interviewer/facilitator | Which author/s conducted the interview or focus group? | 6 |
| 2. | Credentials | What were the researcher's credentials? *E.g. PhD, MD* | 6 |
| 3. | Occupation | What was their occupation at the time of the study? | 6 |
| 4. | Gender | Was the researcher male or female? | Title page |
| 5. | Experience and training | What experience or training did the researcher have? | 6 |
| **Relationship with participants** | | | |
| 6. | Relationship established | Was a relationship established prior to study commencement? | 6 |
| 7. | Participant knowledge of the interviewer | What did the participants know about the researcher? e*.g. personal goals, reasons for doing the research* | 6 |
| 8. | Interviewer characteristics | What characteristics were reported about the interviewer/facilitator? e.g. *Bias, assumptions, reasons and interests in the research topic* | 6 |
| **Domain 2: study design** | | | |
| **Theoretical framework** | | | |
| 9. | Methodological orientation and Theory | What methodological orientation was stated to underpin the study? *e.g. grounded theory, discourse analysis, ethnography, phenomenology, content analysis* | 6 |
| **Participant selection** | | | |
| 10. | Sampling | How were participants selected? *e.g. purposive, convenience, consecutive, snowball* | 5-6 |
| 11. | Method of approach | How were participants approached? e*.g. face-to-face, telephone, mail, email* | 6 |
| 12. | Sample size | How many participants were in the study? | 7 |
| 13. | Non-participation | How many people refused to participate or dropped out? Reasons? | N/A |
| **Setting** | | | |
| 14. | Setting of data collection | Where was the data collected? e*.g. home, clinic, workplace* | 6 |
| 15. | Presence of non-participants | Was anyone else present besides the participants and researchers? | N/A |
| 16. | Description of sample | What are the important characteristics of the sample? *e.g. demographic data, date* | 7, Table 1 |
| **Data collection** | | | |
| 17. | Interview guide | Were questions, prompts, guides provided by the authors? Was it pilot tested? | 6 |
| 18. | Repeat interviews | Were repeat interviews carried out? If yes, how many? | N/A |
| 19. | Audio/visual recording | Did the research use audio or visual recording to collect the data? | 6 |
| 20. | Field notes | Were field notes made during and/or after the interview or focus group? | 6 |
| 21. | Duration | What was the duration of the interviews or focus group? | 6 |
| 22. | Data saturation | Was data saturation discussed? | 6 |
| 23. | Transcripts returned | Were transcripts returned to participants for comment and/or correction? | 6 |
| **Domain 3: analysis and findings** | | | |
| **Data analysis** | | | |
| 24. | Number of data coders | How many data coders coded the data? | 6-7 |
| 25. | Description of the coding tree | Did authors provide a description of the coding tree? | 6-7 |
| 26. | Derivation of themes | Were themes identified in advance or derived from the data? | 6-7 |
| 27. | Software | What software, if applicable, was used to manage the data? | 7 |
| 28. | Participant checking | Did participants provide feedback on the findings? | 6 |
| **Reporting** | | | |
| 29. | Quotations presented | Were participant quotations presented to illustrate the themes/ findings? Was each quotation identified? e*.g. participant number* | 7, Results section |
| 30. | Data and findings consistent | Was there consistency between the data presented and the findings? | Results section |
| 31. | Clarity of major themes | Were major themes clearly presented in the findings? | Results section |
| 32. | Clarity of minor themes | Is there a description of diverse cases or discussion of minor themes? | Results section |

**Table S2:** Interview guide for Meals on Wheels service users

1. Could you tell me how you became aware that Meals on Wheels are available in your area? [**Prompt for**: did someone refer you to the service and, if so, who; how did you find the information you needed, e.g. website, word of mouth, other means?]

[**Follow-up:** Did the people responsible for the MoWs service assess you to see whether you are eligible to receive meals? Can you please describe the process you went through?]

1. How did you come to the decision to use the MoWs service? [**Prompt for**: what motivated you?]

[**Follow-up 1:** How long have you been using MoWs?]

[**Follow-up 2**: Did you use MoWs before the pandemic started?]

1. What information did you want to find out the first time you enquired about MoWs?

[**Follow-up:** What information was given to you?]

[**Follow-up:** Were there other providers of MoWs available in your area, and if so, how did you decide to choose your current provider?]

1. Please describe the meals you receive.

- How often do you receive meals each week, and how many meals per day?
- Do you receive meals at the weekend?
- What time of the day do the meals arrive?
- Tell me about the types of meals you receive.

[**Follow-up 1:** How do you feel about the variety of the meals?] [**Prompt for:** do menus change and how often?]

[**Follow-up 2:** Do you get side dishes, like vegetables, or puddings? Can you describe them?]

[**Follow-up 3:** Do you get to choose what meals you get?] [**Prompt for:** can you tell me about a time when you had a specific meal requirement? How was this accommodated?]

- What do you think of the taste of the meals you receive?
- What do you think of the healthiness of the meals?
- What do you think of the amount of the meals you receive? [**Prompt for:** portion sizes; meals being filling]
- Do the drivers or the MoWs office give you information on how to handle the meals? What information do they give you? [**Prompt for:** re-heating; what to do with leftovers; any other specific instructions?]
- Can you tell me about a time when you were not able to finish your whole meal?

[**Follow up:** what did you do with the leftovers?]

- Do you pay for the meals? What do you think about the cost of receiving MoWs? [**Prompt for:** compared to buying and preparing food from the supermarket or a restaurant; value-for-money?]
- Have you got any suggestions on how the meals could be improved?

Now think of the time before COVID-19 (I will ask you about COVID-19 later)…

1. In what ways do you feel that receiving MoWs benefit you? [**Prompt for**: what do you value about MoWs?]

[**Follow-up:** In what ways do you feel MoWs improve your everyday life? Can you provide examples?]

1. Can you please describe a typical meal delivery interaction with the driver who delivers your meals? [**Prompt for**: how much time does a driver typically spend with you; does a driver provide any assistance to you in addition to handing you a meal, like opening containers, placing in refrigerator, setting up the meal?]
2. Can you please tell me whether the driver provides you with any other assistance, beyond the meal delivery? [**Prompt for:** welfare checks, remind you to take medication, chores around the house, call someone for you; referral to a partnering organisation or social service?]

[**Follow-up 1:** What has your experience been of receiving this assistance?]

[**Follow-up 2:** In what ways do you feel this assistance improves your everyday life?]

1. Can you tell me whether the driver who delivers your meals talks to you at all about physical activity or exercise, and in which context that would be? [**Prompt for:** walking around the house; time spent sitting; gardening?]

[**Follow-up:** How would you feel if they did talk to you about ways to move more around the house? What type of advice would you value?]

1. Does the driver who delivers your meals ever look for safety concerns in your home? What kind of safety concerns do they normally check for? [**Prompt for:** gas leaks; trip hazards; smoke detectors]

[**Follow-up:** Was there a time when they noticed a safety concern? Can you tell me about that? What did they do when they noticed?]

1. Can you tell me about a time when you had to contact the MoWs office directly?

[**Follow-up:** What was your interaction with the office like?] [**REMIND respondent** not to divulge personal information or information that could potentially identify a driver]

1. Can you tell me about a time when you faced a challenge with the meals delivery, a driver or the MoWs service more generally? [**Prompt for:** delay in getting the meals; problem with the meals themselves; feeling unsafe?]

[**Follow-up:** What was that interaction like? How was this challenge overcome?] [**REMIND respondent** not to divulge personal information or information that could potentially identify a driver]

1. Can you tell me about a time when you were referred to other (additional) services by the MoWs service, due to your specific needs or circumstances? [**Prompt for:** classes run in the community; engagement community events]

[**Follow-up 1:** How was this referral made?]

[**Follow-up 2:** Are there additional supportive services that you would like the MoWs service to provide to you? Can you provide some examples?]

1. Now please think of the ongoing COVID-19 pandemic and the national lockdowns that we had in March 2020 and last winter. In what way might your experience with the MoWs service have changed during that time? Can you provide some examples? [**Prompt for:** change in interactions with the drivers; change in interactions with the MoWs office; change in the food delivered; change in any assistance you get from the drivers?]
2. If there was to be a new lockdown in the future, what do you think the implications would be on your experience as a person receiving meals, and your needs from the service?

[**Follow-up:** Do you have any suggestions on how these challenges could be overcome?]

1. Overall, how do you find your experience with the MoWs service?

[**Follow-up:** What things wouldn’t you change and what things you would you change about the service that is provided to you?]

1. Is there anything else you would like to say about your interactions with the MoWs service or the benefits of the service to you that hasn’t been covered?

**Table S3:** Interview guide for Meals on Wheels referrers

1. Could you tell me how you became aware that Meals on Wheels are available in your area? [**Prompt for:** how did you find the information you needed, e.g. website, word of mouth, other means?]

[**Follow-up:** Did the people responsible for the MoWs service assess the person you referred to see whether they are eligible to receive meals? Can you please describe what this involved?]

1. How did you come to the decision to refer a family member, friend or neighbour to the MoWs service? [**Prompt for**: what motivated you?]

[**Follow-up 1:** How long have they been using MoWs?]

[**Follow-up 2**: Did the person you referred use MoWs before the pandemic started?]

1. What information did you want to find out the first time you enquired about MoWs?

[**Follow-up:** What information was given to you?]

[**Follow-up:** Were there other providers of MoWs available in your area, and if so, how did you decide to choose your current provider?]

1. Please describe your experience with the food MoWs provide to the person you referred to the service.

- How often do they receive meals each week, and how many meals per day?
- Do they receive meals at the weekend?
- What time of the day do the meals arrive?
- Tell me about the types of meals they receive.

[**Follow-up 1:** How do you feel about the variety of the meals?] [**Prompt for:** do menus change and how often?]

[**Follow-up 2:** Do they get side dishes, like vegetables, or puddings? Can you describe them?]

[**Follow-up 3:** Do they get to choose what meals they get?] [**Prompt for:** can you tell me about a time when the person you referred had a specific meal requirement? How was this accommodated?]

- What do you think of the taste of the meals they receive? (e.g. has the person you referred told you about the taste or have you tasted the meals yourself?)
- What do you think of the healthiness of the meals?
- What do you think of the amount of the meals? [**Prompt for:** portion sizes; meals being filling]
- Do the drivers or the MoWs office give you or the person you referred information on how to handle the meals? What information do they give? [**Prompt for:** re-heating; what to do with leftovers; any other specific instructions?]
- Who pays for the meals? What do you think about the cost of receiving MoWs? [**Prompt for:** compared to buying and preparing food from the supermarket or a restaurant; value-for-money?]
- Have you got any suggestions on how the meals could be improved?

Now think of the time before COVID-19 (I will ask you about COVID-19 later)…

1. From your experience, or from what the person you referred tells you, what are the benefits of the service to the people who receive the meals? [**Prompt for**: what do clients value about MoWs?]

[**Follow-up:** In what ways do you feel MoWs improve the everyday life of the person you referred? Can you provide examples?]

1. In what ways do you feel that the person you referred receiving MoWs benefits you personally? [**Prompt for**: what do you value about MoWs?]
2. Based on your experience, can you please describe a typical meal delivery interaction between the person you referred and the driver who delivers their meals? [**Prompt for**: how much time does a driver typically spend with the person you referred; does a driver provide any assistance to them in addition to handing them a meal, like opening containers, placing in refrigerator, setting up the meal?]
3. Can you please tell me whether the driver provides the person you referred with any other assistance, beyond the meal delivery? [**Prompt for:** welfare checks, remind them to take medication, chores around the house, call someone for them; referral to a partnering organisation or social service?]

[**Follow-up 1:** What has your experience been of the person you referred receiving this assistance?]

[**Follow-up 2:** In what ways do you feel this assistance improves the everyday life of the person you referred?]

1. Can you tell me whether the driver who delivers meals to the person you referred talks to them at all about physical activity or exercise, and in which context that would be? [**Prompt for:** walking around the house; time spent sitting; gardening?]

[**Follow-up:** How would you feel if they did talk to the person you referred about ways to move more around the house? What type of advice would you value?]

1. Does the driver who delivers meals to the person you referred ever look for safety concerns in their home? What kind of safety concerns do they normally check for? [**Prompt for:** gas leaks; trip hazards; smoke detectors]

[**Follow-up:** Was there a time when they noticed a safety concern? Can you tell me about that? What did they do when they noticed?]

1. Can you tell me about a time when you personally had to contact the MoWs office directly?

[**Follow-up 1:** What was your interaction with the office like?] [**REMIND respondent** not to divulge personal information or information that could potentially identify a driver]

[**Follow-up 2:** Can you tell me about a time when you were worried about the person you referred and you had to notify the MoWs office?] [**Prompt for:** what was done with this information? What happened next?]

1. Can you tell me about a time when you or the person you referred faced a challenge with the meals delivery, a driver or the MoWs service more generally? [**Prompt for:** delay in getting the meals; problem with the meals themselves; feeling unsafe?]

[**Follow-up:** What was that interaction like? How was this challenge overcome?] [**REMIND respondent** not to divulge personal information or information that could potentially identify a driver]

1. Can you tell me about a time when the person you referred was referred to other (additional) services by the MoWs service, due to their specific needs or circumstances? [**Prompt for:** classes run in the community; engagement community events]

[**Follow-up 1:** How was this referral made?]

[**Follow-up 2:** Are there additional supportive services that you would like the MoWs service to provide to you and the person you referred? Can you provide some examples?]

1. Now please think of the ongoing COVID-19 pandemic and the national lockdowns that we had in March 2020 and last winter. In what way might the experience of the person you referred with the MoWs service have changed during that time? Can you provide some examples? [**Prompt for:** change in interactions between the person you referred and the drivers; change in interactions between the person you referred and the MoWs office; change in the food delivered; change in any assistance the person you referred got from the drivers?]

[**Follow-up:** In what way might your own personal experience with the MoWs service have changed during the pandemic? Can you provide some examples?]

1. If there was to be a new lockdown in the future, what do you think the implications would be on the experience of the person you referred as a person receiving meals, and their needs from the service?

[**Follow-up 1:** Do you have any suggestions on how these challenges could be overcome?]

[**Follow-up 2:** What do you think the implications would be on your own personal experience of the MoWs service? Do you have any suggestions on how these challenges could be overcome?]

1. Overall, how do you find your experience with the MoWs service?

[**Follow-up:** What things wouldn’t you change and what things would you change about the service that is provided to the person you referred?]

1. Is there anything else you would like to say about your interactions with the MoWs service or the benefits of the service to you or the person you referred that hasn’t been covered?

Table S4: Additional quotations from the themes and sub-themes resulting from the thematic analysis

| **Theme** | **Sub-theme and quotations** |
| --- | --- |
| **Accessing and commencing the service** | **- Referrals to Meals on Wheels**  Professional referrals:  *‘My eye surgeon, they made a report to [] Council and they sent a woman out to see what I needed. She gave me the address of it. Because my wife is dead now and I can’t cook anymore because I can’t see. That’s how I got the address of [service provider]. This is the only option I was given at the time. Because I’m housebound more or less now and because I can’t read anymore, I don’t know any better and I’m quite satisfied with this service anyway’. (SU1)*  *‘Well, it was my carer… she was coming in every week and if there was anything I wanted to know about, she usually rang up for me.. So, she really has become a very, very strong link in getting me sorted with outside services…’ (SU2)*  *‘I had a care needs assessment, and that was carried out by a social worker from the Council. She came and did a thorough investigation into what my care needs were now, at the time… She said she would do it for me. I think you can refer yourself to the meals on wheels service, but she would do it for me, and she did.’ (SU3)*  *‘I think somebody told me, I can’t remember. Somebody else that I know who was elderly they said they had Meals on Wheels. It was in a conversation with somebody, I can’t remember who it was now. But they said, “Oh, have you tried Meals on Wheels” so I thought I’d look in to it and asked my support worker to look in to it for me. She said, “Well, I’ll set you up.” She helped me fill out the form and everything and she put me in touch with them. They rang me and said, “Yeah, you’re good to go.”…You can look at the menu and see what you want, tick all the boxes of the one you want and then they give it to you’. (SU5)*  *‘I'm not sure if they're from the hospital, they're attached to the hospital, but they're very much domiciliary care in the old fashioned expression…They certainly told me of its existence.’ (R1)*  *‘Well, I’ve known about Meals on Wheels for a long time. My mum died a long time ago, but she was a volunteer in the old system. So, we knew Meals on Wheels as a concept, but we only started to need it after Dad had severe problems and we realised what it was, and we immediately- well, the social worker came and we thought, “Yes, this would be an excellent thing to put in place for him.” (R9)*  *‘It was actually the Social Services people in [name of council] who referred the patient to- Well, we discussed it and it was agreed that he was no longer able to prepare his own food but he needed a hot meal every day, so that was deemed to be the most appropriate way of supporting him to stay in his own flat, if you like’ (R11)*  *‘…I think actually the pack came from adult social care as part of the programme that she was going to be introduced into. So, for example, when they had people coming into the house to see if she needed any physical assistance, they gave us a pack in terms of if there’s anything that maybe the community could help with. So, essentially, just a pack that came from social care, just what might be beneficial to us as a family, as such.’ (R12)*  *‘The social services I spoke to about the Meals on Wheels was the [name of council] social services who were absolutely excellent’ (R14)*  *‘And I think their social workers were involved so I think they suggested it as well and then I actually followed it up.’ (R17)*  Referrals by family members  *‘I looked it up online, because she’s 94 and I knew that she wasn’t cooking. So I just looked it up, to see what existed’ (R2)*  *‘No, I set them up. She had just come out of hospital as well. So she was getting a reablement package through the council. So I think they were aware that she would need them, but then when I contacted them, no one else referred, so I did it myself’(R4)*  *‘I think I've always known about Meals on Wheels (Laughter) for many years. It wasn't until I was in a position where I had my mother who then needed some support. So then I phoned the council, funnily enough, because I wasn't really sure who provided them. So I called the council and they were really good. They gave me all the information, gave me the phone number, and went direct from there’ (R4)*  *‘Ironically my mum has other health issues, she has Diabetes Type II, and she needed to have- I had to- she wasn’t taking medication and all the rest of it and so she ended up needing a district nurse every day… Anyway, so it all came about through that. So I was referred to [name of council] to sort it all out and then on their website came up the advert for community meals and all that. So I’ve always been aware that that’s something in my toolbox, if you like, that I might have to put in place for one or other of them’ (R5)*  *‘So it was just looking to see what was available to help me and my brothers to support the two of them because we don’t all live very, very close to them. So it was seeing what resources there were out there, that’s how I came across community meals’ (R5)*  *‘Yes, I’ve always worked in health and social care up until recently. So, I know of clients that I’ve had in the past that have used a service locally to me. So, all I did was went on Google and looked at some services in the [] area and looked at reviews, prices, locality and what options they offered, and basically, took it from there…’ (R6)*  *‘I called them when we were trying to sort out her meal options of what she did and didn’t like. Yes, the girls in the office who pick up the phone were always really helpful, really friendly. Yes, can’t fault them at all.’ (R8)*  *‘After he was diagnosed, he had a full assessment, but we’re self-funders. It sounds a bit strange, but we’re knowledgeable people and I decided it’s something that I would look into and I just asked around, found out what was available. I think had we been spending the authority’s money, it might have been different, but we were spending our own. So, we felt like customers, and that’s how I approached it’ (R9)*  *‘I had a look at what was available in our area, and then I phoned. They sent me links to their more detailed website. The girls in the office were really helpful, really friendly. They just seemed like a good company to go with. So then I did some research, found Meals on Wheels, found one that covered our area, and we’ve been using them ever since.’ (R10)*  *‘I went online, and I just did a bit of a Google search and then I got quite a bit- their information was quite good. The information they have online I found quite interesting. It’s like a PDF document that had lots of information in there. I sent it to my dad to have a look at as well, but it was- yeah, it was pretty informative. I did give them a ring just to double check on a few things. But yeah, that’s what I found out about it’. (R15)*  *‘He wouldn’t have gone off his own back to do it. It was only that I thought that perhaps it’s a good- “Well let’s give it a go dad” and he was like, “Oh yeah, let’s give that a go.” But yeah, I’m not sure that he would have thought about that himself.’ (R15)*  *‘It’s a really difficult situation really. Mental health have been involved from [name of council] and the doctor is involved. She’s very difficult, really difficult, but, no, it was done by me, I rang meals on wheels really…’ (R16)*  *‘Well, I rang the lady. I have found them very good I have to say. I rang and spoke to the lady on the end of the phone who deals with menus and things like that. And she went through a lot of things with me’ (R16)*  *‘I think, yes, I went online, yes. I just Googled, ‘Meals on Wheels’ and something came up, and there was a number from that I actually phoned through (R19)*  *‘Well, so we did have a brochure. I think my partner rang them up and requested the brochure sent to my mother. But, yes, that was the main thing’ (R21)*  **- Knowledge of the MoWs concept and commencing the service**  *‘Yes, they gave me a booklet as well, which was pretty- I couldn’t read it but she did explain what I have to do. It’s really straightforward actually.’ (SU1)*  *‘…that girl I was talking about, she likes to fill that in when she comes because I can’t. They give you a daily menu sheet, a weekly menu sheet, I should say, and you tick the boxes which you want and then one of the Meals on Wheels people pick it up and take it to their headquarters and that’s delivered the following week. It’s a bit like being in hospital, if you know what I mean, and you fill the thing in for what you want for tomorrow, if you’ve ever been in hospital’. (SU1)*  *‘…there’s a telephone number that they sent me initially and I keep it… It was written on a nice, big, bright yellow card with the details of the service and the telephone number and asking, meal service, 'Please contact us before 9:00am if you wish to cancel your meal, otherwise you will be charged.' So, it was really clear cut, as to the rules that they work by’. (SU2)*  *‘So the community meal service was very clear and it’s quite a simple concept... even if it’s not called Meals on Wheels anymore, we always used to call it that- and I think it’s quite a well-known concept, as it were.’ (R3)*  *‘Yes, the first day they came, they gave me a welcome pack, and it had quite a lot of information in it about the service, about the cost, and three weeks menus, so we could order in advance… So every three weeks, they send the next three weeks. It's a rolling three-week menu with three or four different choices. So then we just tick them off and give them back when they come’. (R4)*  *‘They can choose from a relatively brief menu, but it’s a menu nevertheless, what they most like. So that’s how we did it at the beginning, I sat down with my aunt and we ticked the sorts of things that she likes. I did the same with my mum’. (R5)*  *‘It is really easy to set up and the two gentlemen that I deal with, the two managers, they’ve been brilliant.’ (R6)*  *‘Obviously always been aware of the service for years, but at Christmas my Nan suffered a mini stroke and so when she came out, she’s okay and she can obviously do things, but using the cooker and getting someone just coming in to check on here, that’s when we started to use the Meals on Wheels service’. (R8)*  *‘I’ve known about Meals on Wheels for a long time because my mum used to deliver Meals on Wheels, when it was run by the Women’s Royal Voluntary Service… And as a child, I used to go and help her deliver. So I knew it was out there, but I didn’t know what format it was in nowadays’. (R10)*  *‘Well, vaguely aware about it probably- Crumbs, I don't know, always really. But personally involved with it, since just after the first lockdown, which was what, 2019?’ (R11)*  *‘Oh, yes, yes. Well, I’m sure that I must have heard of it in terms of maybe in the media or possibly, you know, just talking to other people as such, but it’s not something that you register. I mean, I’m sure I’ve heard the name ‘Meals on Wheels’ before, but until you really experience it, you don’t really know what it’s about’. (R12)*  *‘It’s actually quite straightforward. You know, it’s quite easy, it’s quite intuitive how the system works. Once you order a few times, it’s really straightforward. So, yes, I’ve got everything that I needed from the Meals on Wheels in our area once I signed up. So, yes, it was quite easy.’ (R12)*  *‘Erm, well I’ve probably always known about it really. To be fair probably even from perhaps my nan’s- when my nan used to have a Meals on Wheels service. So I’ve always been aware that there is a Meals on Wheels service. Yeah, so that’s probably where it’s come from historically through the family probably’. (R15)*  *‘I would say it’s very good and I would recommend it to other people. I haven’t really had any issues. I think organising it was pretty straight forward’. (R15)*  *‘Well, I used to live in [name of city], so I always knew Meals on Wheels was available to elderly people’. (R17)*  *‘That’s the only way I knew about it, is my mother knew, but I don’t know, how did she find out? Oh, I don’t know, but she talks to people, so I would guess that it’s word of mouth, really, I would suggest’. (R19)*  *‘Yes, pretty good actually; whoever it was I spoke to, sorry, I don’t know their name, they were excellent, they were very good, very quickly sorted it out, actually. Very efficient I thought, yes, very good’. (R19)*  *‘It's been very straightforward, very easy to speak with, and to be honest, yes, very straightforward and very easy to deal with, yes’. (R21)*  *‘Yes. I think the website is very accessible and certainly whoever you speak to at the end of the website is very good.’ (R22)*  **- Barriers to accessing Meals on Wheels**  *‘My neighbour, I introduced her to it and she’s in her 80s now, and she was housebound and she couldn’t… So, I said, “Well, why don’t you get yourself a worker too, to give you the meals on wheels?” and she said that it reminded her of school meals, but I mean school meals, I had them when I was a child. They were brilliant, yes… So, yes, I know a lot of people that are a bit snobby about this, but I have really good meals, tasty meals…’ (SU3)*  *‘And when I suggested it to mum in the autumn, it was like I’d suggested something for old people, and she pooh-poohed it, and she looked at me like I’d suggested I don't know what.’ (R2)*  *‘This sounds longwinded, but it’s relevant. When I said, after she came home, “Mum, you’ve just been in hospital, I think it would be a really good idea if we did- Just try it for a week, these meals.” And she agreed to do it. Now, it has gone really well, as I’ll tell you, and we’ve just not stopped. She thought initially it was some sort of convalescence thing [anon] had organised and she’d just go along with it, you know?’ (R2)*  *‘Well, first of all, she was very resistant to it, very resistant, because Meals on Wheels are for old people. (Laughter) She's 88, bless her.’ (R4)*  *‘… he doesn’t want to and he says, you know, he’s fine and he’ll make do, but obviously, he’s my dad and I need him to have a hot meal’. (R5)*  *‘… there’s a, sort of, stereotype with Meals on Wheels that it is only for old people who can’t get about, as in mobility, and it’s not that at all. I think that stereotype needs to be taken away. The only way that it will be taken away is for people to be given different information, and especially, like I said, when I looked on the internet and saw you get a lot of… you know when they put illustrations in the corner of the webpage and it’s old people. It’s really old people and it’s marketed at old people.’ (R6)*  *‘I mean, my dad’s 57. He would never even have thought of Meals on Wheels, and when I suggested it to him, he went, “No, it’s for old people, [anon],” and I went, “It’s not. It’s for anybody who has a need for a hot meal who physically can’t do it themselves.”’ “No, it’s not, it’s not,” and I said, “It is. Let’s just trial it for a couple of weeks and see how we get on.” They were like, “This is not bad, this, is it?” It’s a stereotype, it’s for old people, and it’s really not.’ (R6)*  *‘Yes, and the stigmatism that used to be, “Ooh, got Meals on Wheels?” because they seem to have come up, the quality of their food and everything, I don’t think that exists anymore. Not that we’d really care if it did, but yes, I think it’s got quite a good reputation for itself now.’ (R8)*  *‘Yes, so my Nan always used to turn her nose up at the thought of Meals and Wheels because I think someone in your family had them years ago, didn’t they, and they were quite bad quality then? Trying to convince her to have Meals on Wheels in the first instance, it was a little bit of a struggle, but after… her stroke, we opted for it and I think she was pleasantly surprised with the quality of it and obviously someone coming in and not having to cook anymore.’ (R8)*  *‘Well, what I did, because Dad was quite reluctant because he perceived it as something that he shouldn’t have to do, so what I wanted, and I ordered at first for two or three weeks, meals for myself, Dad and my sister. So, all three of us sat down and had the meals, and it was a way of introducing Dad.’ (R9)*  *‘The lady who is receiving it used to give meals on wheels herself, she used to do it voluntarily before her Alzheimer’s years ago. And, so, we found that that was the best method of doing it with her because she could relate to the fact that she’s done it herself… But what was causing the problem for me was because she had done it herself, she didn’t consider herself old enough to be receiving it even though she’s 79 at the moment, nearly 80’. (R16)*  *‘So it’s obviously quite hard to get – and I’m guessing here - I assume from my mother it’s quite difficult to get the elderly to do this because, “They don’t need the help apparently”, as I’m constantly being told’. (R21)* |
| **Information valued when enquiring about MoWs for the first time** | **- Information relating to the meals**  *I just wanted to know what type of meals are provided, the cost and whether they provided it regularly without interruption and they have done that. (SU1)*  *I wanted to find out what they provided. Again, whether it was a fully prepared hot meal that he simply needed to eat, or whether there was any preparation involved. Because at that time, I suspected he couldn't be bothered preparing it. So that was the first thing. (R1)*  *What I wanted to ensure was that he got a cooked meal that he just had to eat. So they told me all about it. I questioned them. It sounded promising. (R1)*  *‘So, I know of clients that I’ve had in the past that have used a service locally to me. So, all I did was went on Google and looked at some services in the (name of area) area and looked at reviews, prices, locality and what options they offered, and basically, [took it from there. I personally look at the reviews, and actually, with this company that we’ve chosen, there weren’t that many reviews, but I phoned up and spoke to, I think it’s the contracts manager and then the actual manager, I spoke to him also and just, sort of, quizzed them about what they could offer. Not in the internet, no. I had to ring them. A lot of companies tend not to put the prices on. So, you do have to ring to get the prices, which I found very frustrating because, you know, for me personally, I didn’t have time to do all of that. We needed a quick-fix there and then. It was, sort of, the choice between either that it has a hot meal or nothing at all, and so for me, I’m a single parent with two children and work full-time. I wanted everything to be there, information-wise. Nutritional information wasn’t there either. So, that was a big thing for me.’ The only way that it will be taken away (the MoWs stigma) is for people to be given different information, and especially, like I said, when I looked on the internet and saw you get a lot of… you know when they put illustrations in the corner of the webpage and it’s old people. It’s really old people and it’s marketed at old people. So, I think that because they’re, sort of, marketing it at older people, people don’t understand what is out there and what is available, and that it’s not just companies just mass-producing, they’re just for profit. I think they could sell themselves a lot better than what they do, but also, I think, like I mentioned to you earlier, something needs to be available for those that have got a primary healthcare need that are unable, physically and mentally, to make and prepare themselves a warm meal, because there’s people going without any food (R6)*  *My brother lives in a retirement apartment, there about 15 years. We had the kitchen refitted when he moved into it and the oven has never been used. He simply cannot use the oven. It has to be microwave. I had to make sure that their meals were microwaveable, let’s put it that way. Also, I needed a degree of flexibility as well. (R7)*  *For her to get a daily balanced hot meal. (R10)*  *‘I wanted to know that they’d got a varied and balanced menu. I wanted to know that the meal would be delivered at a time that my mum would think is suitable for lunchtime. And I just wanted somebody that wouldn’t have a lot of change of staff. Because of her Alzheimer’s, I wanted pretty regular faces turning up at her door, really.’ (R10)*  *Well, I was clearly concerned about things like the nutritional value and that kind of thing. And my understanding was, and still is, that it’s designed to be nutritionally-balanced. (R11)*    *So, I wanted just there to be somebody who could provide a hot meal that she could eat there and then, you know, and then that would be having some hot food in her tummy, sort of thing. So, that probably would be the most important thing for me, but also choice, you know, that they have some nice choices, so yes. (R12)*  *My gut-feeling was to make sure that when I wasn’t there, my mum was going to eat something that was warm, and as they say, healthy, nutritious and tasty. So, obviously, that’s important because sometimes, the ready-meals, you’re thinking, “Ah, too much salt, too much....” They’re not always good, and the fact that it would be served in front of her. (R12)*  *I suppose it would be the, sort of, regularity of the meals and the choices, and, of course, the cost. That has to be factored in. I do have power of attorney for my mum. So, I need to be careful, you know, her budget, if she’s okay with her money, but I think the most important thing for me would be in terms of the choices. (R12)*  *Well it’s what’s important to the lady with Alzheimer’s and principally that- well, in equal order really, that it’s a nutritious meal that she was not getting before with the carers she had, that it’s a regular time because consistency is very important to someone with Alzheimer’s, and that’s it’s a personal service. Because she is not able to- well she can but not reliably put it on a plate. So I needed to know that someone would go into the house, put it out for her and make sure she was happy and make sure she had a drink. So those three are really very important because they’re the three areas that cause the biggest problems. (R13)*  *‘It was important to know that it was a good diet, the meals themselves, you know, they’re getting a varied diet. So, that was important.’ (R14)*  *Well again, because my daughter actually started it, we just wanted to make sure that they could both have a hot meal during the day. And also, because my dad is a very fussy eater and Meals on Wheels were brilliant, you just altered the menu for my dad, which was lovely as well, so, which was really good… there’s only certain foods that he’d eat, he’d eat like fish or like omelettes and things like that but again which they catered for Dad, so he was okay (R17)*    **- Information relating to the specific services provided**  *I just wanted to know what type of meals are provided, the cost and whether they provided it regularly without interruption and they have done that. (SU1)*  *‘Well, basically how much it was going to cost because obviously with the cost of living and everything nowadays.’ (SU6)*  *… Very helpful. Very forthcoming, very down to earth. So I rang him and chatted at some length about how it all worked and satisfied myself that it was probably what we wanted and that it was flexible enough that if I go up to visit [anon], which I try to do as often as I can, you can cancel a given day's meal as late as 10:00 that morning. So that's tremendously flexible. (R1)*  *‘I wanted to make sure that whoever came in every day, and I know I feel absolutely safe all the time when the community meals people come in, that there’s that level of integrity for safeguarding for adults as well.’ (R3)*  *I needed to know that- what really appealed to me was the fact that it was another set of eyes. I couldn’t see mum and [anon] everyday although we tried video contact with Messenger. That sort of works. But what sold me on it was the fact that they would be happy to report back if they felt there was something different that they weren’t happy with. Something about this person they see every day. They would be going in just briefly and they would notice if there was a problem, and they would then feed that back to the head office who would then feed that back to me. So it was important to me to know that there was just another person just popping in, albeit for a very brief time, that could effectively tell me if there was a problem that they felt was going on. That’s what sold me on it other than the fact that it was a hot meal for somebody who wasn’t bothering to cook for herself. (R5)* |
|  | *For me and my mum and my auntie, more of someone coming in every day. The food, obviously I was assuming that it would have been good quality because of obviously the people that they would serve it to, but probably more it was the fact that someone was coming every day to just check and say hello. (R8)*  *Touch wood, if anything was wrong, that they would also obviously contact us, where other services for home meal delivery, they all give you a week’s worth of food in one day. You haven’t got someone daily coming in. Yes, so a hot meal. She doesn’t have to cook herself, doesn’t have to use the oven, can’t do any damage to herself, but also someone coming in every day (R8)*  *… and we were looking for quality, reliability and friendly people, people that Dad could feel… you know, his pride wasn’t suffering. That’s what I was looking for. (R9)*  *‘I wanted to know that they’d got a varied and balanced menu. I wanted to know that the meal would be delivered at a time that my mum would think is suitable for lunchtime. And I just wanted somebody that wouldn’t have a lot of change of staff. Because of her Alzheimer’s, I wanted pretty regular faces turning up at her door, really.’ (R10)*  *I suppose it would be the, sort of, regularity of the meals and the choices, and, of course, the cost. That has to be factored in. I do have power of attorney for my mum. So, I need to be careful, you know, her budget, if she’s okay with her money, but I think the most important thing for me would be in terms of the choices. (R12)*  *Well it’s what’s important to the lady with Alzheimer’s and principally that- well, in equal order really, that it’s a nutritious meal that she was not getting before with the carers she had, that it’s a regular time because consistency is very important to someone with Alzheimer’s, and that’s it’s a personal service. Because she is not able to- well she can but not reliably put it on a plate. So I needed to know that someone would go into the house, put it out for her and make sure she was happy and make sure she had a drink. So those three are really very important because they’re the three areas that cause the biggest problems. (R13)*  *The driver comes in. He’s very, very friendly. He’ll just tap on the window. She lets him in. He’ll go straight through to the kitchen and plate it up for her. One of the big factors that I checked on when I made the original enquiries was it’s not just a drop, dash and run, is it? I wouldn’t like someone who goes in and just is politely saying, “Are you alright love? Any problems?” and if he’s happy, then he can go. If there’s a concern, would they let me know? But the answer is yes, and I’m very happy with it (R13)*  *‘So I called them because I just wanted to know more information about how you pay to start off with because I think it started off we could pay as we go weekly for a bit. We’re on direct debit now. Because if it was going to be temporary, I wanted something easy to set up so that we could cancel it if we wanted to. I wanted to check about- do you need to have every day, could you take some days off, so that was important to us. Yeah, I think probably the flexibility of it really was probably important to start off with. Because, like you say, our thinking was that it was going to be temporary rather than somewhere that got to a point where it was essential for them, if you know what I mean?’ (R15)*  *Okay, well, she’s not mobile, really, so she can't answer the door, in fact, so we needed somebody who could actually bring the meals in, prepare, at least prepare them in a way that they’re ready for her to access, and then basically sit down and eat. And also possibly offer her some help in terms of, well okay, if necessary, get her a drink and, well, she can move but she has to use the frame, so therefore we need to have – it wasn’t a case of just turning up, dumping it on the doorstep and going away, that would’ve been completely inappropriate – it had to be somebody who would actually come into the house, and say, “Okay, here’s your meals, here they are.” (R19)*  *‘So one of the things that was quite important is the time they turned up was a regular time. It also was very important they’d have if not the same person every day, at least regulars if you see what I mean?’ (R21)*  *The price obviously is very important. Ridiculously cheap really. (R21)* |
